# Genetics and intelligence differences: five special findings

**DOI:** 10.1038/mp.2014.105

**Published:** 2014-09-16

**Authors:** R Plomin, I J Deary

**Affiliations:** 1 King's College London, MRC Social, Genetic & Developmental Psychiatry Centre, Institute of Psychiatry, DeCrespigny Park, London, UK; 2 Department of Psychology, University of Edinburgh, Edinburgh, UK; 3 Centre for Cognitive Ageing and Cognitive Epidemiology, University of Edinburgh, Edinburgh, UK

## Abstract

Intelligence is a core construct in differential psychology and behavioural genetics, and should be so in cognitive neuroscience. It is one of the best predictors of important life outcomes such as education, occupation, mental and physical health and illness, and mortality. Intelligence is one of the most heritable behavioural traits. Here, we highlight five genetic findings that are special to intelligence differences and that have important implications for its genetic architecture and for gene-hunting expeditions. (i) The heritability of intelligence increases from about 20% in infancy to perhaps 80% in later adulthood. (ii) Intelligence captures genetic effects on diverse cognitive and learning abilities, which correlate phenotypically about 0.30 on average but correlate genetically about 0.60 or higher. (iii) Assortative mating is greater for intelligence (spouse correlations ~0.40) than for other behavioural traits such as personality and psychopathology (~0.10) or physical traits such as height and weight (~0.20). Assortative mating pumps additive genetic variance into the population every generation, contributing to the high narrow heritability (additive genetic variance) of intelligence. (iv) Unlike psychiatric disorders, intelligence is normally distributed with a positive end of exceptional performance that is a model for ‘positive genetics’. (v) Intelligence is associated with education and social class and broadens the causal perspectives on how these three inter-correlated variables contribute to social mobility, and health, illness and mortality differences. These five findings arose primarily from twin studies. They are being confirmed by the first new quantitative genetic technique in a century—Genome-wide Complex Trait Analysis (GCTA)—which estimates genetic influence using genome-wide genotypes in large samples of unrelated individuals. Comparing GCTA results to the results of twin studies reveals important insights into the genetic architecture of intelligence that are relevant to attempts to narrow the ‘missing heritability’ gap.

## Introduction

Nearly a century ago, intelligence was the first behavioural trait studied using newly emerging quantitative genetic designs such as twin and adoption studies.^1, 2, 3, 4^ Such studies have consistently shown that genetic influence on individual differences in intelligence is substantial.^5,6^ Intelligence has become the target of molecular genetic studies attempting to identify genes responsible for its heritability.

Here, we refrain from providing another general overview of the genetics of intelligence. We begin by noting three regularities that might almost be dubbed ‘laws’ from genetic research that apply to many traits in the life sciences. The bulk of our review highlights genetic findings that are specific to intelligence rather than these general laws.

## Three ‘laws’ of the genetics of complex traits (including intelligence)

### All traits show significant genetic influence

Finding that differences between individuals (traits, whether assessed quantitatively as a dimension or qualitatively as a diagnosis) are significantly heritable is so ubiquitous for behavioural traits that it has been enshrined as the first law of behavioural genetics.^7^ Although the pervasiveness of this finding makes it a commonplace observation, it should not be taken for granted, especially in the behavioural sciences, because this was the battleground for nature-nurture wars until only a few decades ago in psychiatry,^8^ even fewer decades ago in psychology,^9^ and continuing today in some areas such as education.^10,11^ It might be argued that it is no longer surprising to demonstrate genetic influence on a behavioural trait, and that it would be more interesting to find a trait that shows *no* genetic influence.

### No traits are 100% heritable

For some areas of behavioural research—especially in psychiatry—the pendulum has swung so far from a focus on nurture to a focus on nature that it is important to highlight a second law of genetics for complex traits and common disorders: All traits show substantial environmental influence, in that heritability is not 100% for any trait. Acceptance of the importance of both genetic and environmental influences leads to interest in the interplay between genes and environment, such as their interaction (moderation) and correlation (mediation) in the development of complex traits, Plomin *et al.*^6^ pp 105–127.

### Heritability is caused by many genes of small effect

The first two laws come from quantitative genetic research, which uses, for example, the twin method to assess the net contribution of genetics to individual differences without knowledge of the genetic architecture of a trait, such as the number of genes involved or their effect sizes. A third law has emerged from molecular genetic research that attempts to identify specific genes responsible for widespread heritability, especially genome-wide association (GWA) studies of the past few years: The heritability of traits is caused by many genes of small effect.^12^ This was the premise of quantitative genetic theory set out nearly a century ago,^13^ but quantitative genetic methods themselves could not shine much light on the distribution of the effect sizes of genes in the population. For decades, the failure of linkage analyses to identify replicable linkages to chromosomal regions could be interpreted as support for this hypothesis because linkage has little power to detect small effect sizes. However, GWA studies have made it clear that the largest effect sizes of associations are very small indeed. For example, we are aware of almost no replicated genetic associations that account for more than 1 per cent of the population variance of quantitative traits such as height and weight. Because GWA studies have adequate power to detect such effect sizes, we can conclude that there are no larger effect sizes, at least for the common single-nucleotide variants that have been used in such studies to date. If the largest effect sizes are so small, the smallest effect sizes must be infinitesimal, which means that such associations will be difficult to detect and even more difficult to replicate. For example, the largest GWA study of intelligence differences, which included nearly 18 000 children, found no genome-wide significant associations. The largest effect sizes accounted for 0.2% of the variance of intelligence scores.^14^ Another recent GWA study of a sample of 1500 children reported an association that accounted for 0.5% of the variance of intelligence scores,^15^ but this association showed no effect in the study of 18 000 children (*P*=0.73; Benyamin B, personal communication). A GWA of educational attainment—which correlates moderately with intelligence—included more than 125 000 individuals; the DNA variant with the largest effect size accounted for 1% of the variance in years of education but the variance explained was only 0.02% in a replication sample.^16^ ‘Missing heritability’ is the catch-phrase to describe the great gulf between heritability and the variance explained by associations with specific DNA variants.

Rather than reviewing evidence for these general laws in relation to intelligence, our review focuses on five findings from genetic research that are specific to intelligence. Because of the controversy and confusion that continues to surround intelligence, especially in the media and the general science literature,^11^ we begin by briefly discussing the definition, measurement and importance of intelligence.

## What is intelligence and why is it important?

Although there are many types of cognitive ability tests of individual differences, they almost all correlate substantially and positively; people with higher ability on one cognitive task tend to have higher ability on all of the others. Intelligence (more precisely, *general cognitive ability* or *g,* as discovered and defined by Spearman in 1904^17^) indexes this covariance, which accounts for about 40 per cent of the total variance when a battery of diverse cognitive tests is administered to a sample with a good range of cognitive ability.^18,19^ As long as a battery of cognitive tests is diverse and reliable, a general ‘factor’ (often represented by the first unrotated principal component, which is not strictly a factor, but that is the terminology that is often used) indexing intelligence differences will emerge and correlate highly with such factors derived from other batteries using wholly different cognitive tests.^20^ The general intelligence component (factor) is a universally found statistical regularity, which means that some have tried to provide an epithet for what it might capture. According to one view, the core of this general intelligence factor is ‘the ability to reason, plan, solve problems, think abstractly, comprehend complex ideas, learn quickly, and learn from experience’ (Gottfredson *et al.*^21^ p.13; see also Deary^22^). Intelligence is at the pinnacle of the hierarchical model of cognitive abilities that includes a middle level of group factors, such as the cognitive domains of verbal and spatial abilities and memory, and a third level of specific tests and their associated narrow cognitive skills.^18,23^

Intelligence is important scientifically and socially. Because intelligence represents individual differences in brain processes working in concert to solve problems, it is central to systems approaches to brain structure and function,^24, 25, 26^ and to the conceptualisation of how diverse cognitive abilities decline with age.^27^ It is also one of the most stable behavioural traits, yielding a correlation of 0.63 in a study of people tested at age 11 and then again at age 79.^28^ Socially, intelligence is one of the best predictors of key outcomes such as education and occupational status.^29^ People with higher intelligence tend to have better mental and physical health and fewer illnesses throughout the life course, and longer lives.^22,30^

The rest of this review describes five genetic findings that are special to intelligence differences: dramatic increases in heritability during the life course, high genetic correlations among diverse cognitive abilities, high assortative mating, the positive genetics of high intelligence and the impact of intelligence on ‘social epidemiology’. Most of these findings are not new,^31^ but highlighting these findings as special for intelligence is novel. Moreover, support for these findings has increased in recent years from traditional quantitative genetic research using the twin design that compares identical and fraternal twins, and, importantly, from a new quantitative genetic method that uses DNA alone to estimate overall genetic influence in large samples of unrelated individuals. This method, which we will refer to as Genome-wide Complex Trait Analysis (GCTA),^32, 33, 34, 35^ is the first new human quantitative genetic method in a century, and is described in Box 1.

### Heritability increases dramatically from infancy through adulthood despite genetic stability

It would be reasonable to assume that as we go through life, experiences—Shakespeare’s ‘whips and scorns of time’—have a cumulative effect on intelligence, perhaps overwhelming early genetic predispositions. However, for intelligence, heritability increases linearly, from (approximately) 20% in infancy to 40% in adolescence, and to 60% in adulthood. Some evidence suggests that heritability might increase to as much as 80% in later adulthood^47^ but then decline to about 60% after age 80.^48^

**Figure 1:**
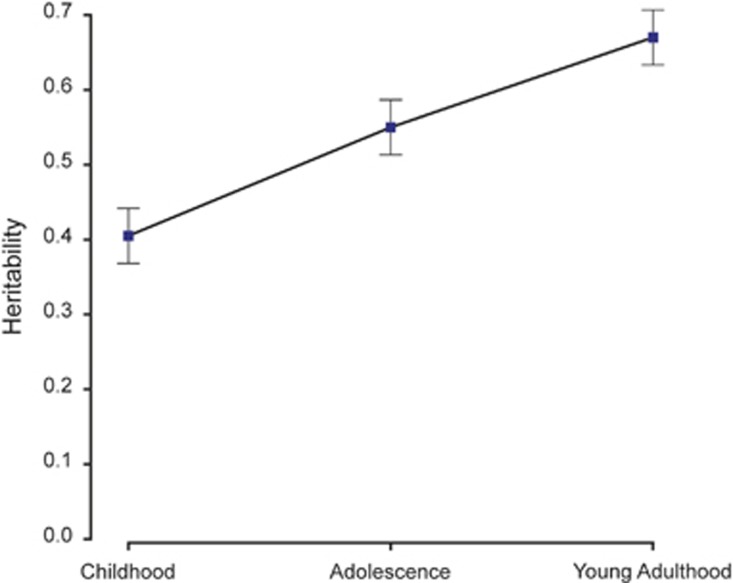
A meta-analysis of 11000 pairs of twins shows that the heritability of intelligence increases significantly from childhood (age 9) to adolescence (age 12) and to young adulthood (age 17). (Adapted from Haworth *et al.*^49^).

Most genetic research has been consistent with this dramatic increase in heritability for intelligence in the early human life course. Figure 1 shows the results of the first study to demonstrate significant increases in heritability in cross-sectional analyses of 11 000 twin pairs from childhood (~40%) to adolescence (~50%) to young adulthood (~60%).^49^ The non-overlapping standard errors across the three ages indicate that the increases in heritabilities are significant. Although these findings have been criticised because they rely on cross-sectional comparisons (Mackintosh^50^ p. 278), similar results showing increases in heritability have been found in longitudinal adoption studies^51,52^ as well as in longitudinal twin studies from early to middle childhood^53,54^ and from middle childhood to adolescence.^55^ Although GCTA can be used to test this finding of increasing heritability across development, the first two attempts to do so using longitudinal data did not have sufficient power to detect the hypothesised age differences in GCTA heritability. One study reported an increase in GCTA heritability of intelligence from 0.26 (0.17 standard error) at age 7 to 0.45 (0.14) at age 12.^56^ Another study reported a decrease in GCTA heritability from 0.48 (0.18) at age 11 to 0.28 (0.18) in old age.^46^ Given the differences in the ages tested in these latter two studies, they are not directly comparable. As indicated by the large standard errors, larger longitudinal studies are needed.

Why does the heritability of intelligence increase so dramatically from childhood to adulthood, as seen in twin studies? A clear yet apparently contradictory finding constrains possible answers to this question. Despite this great increase in heritability, the same genes affect intelligence from age to age. For example, a recent twin study reported a genetic correlation of 0.75 (standard error=0.08) from age 7 to age 12, despite increasing heritability from 0.36 (0.03) to 0.49 (0.04) and despite mean changes in brain structure and function from childhood to adolescence.^55^ GCTA analyses in the same study but using unrelated individuals yielded a highly similar genetic correlation of 0.73 (0.29) from age 7 to age 12. Most strikingly, a 60-year longitudinal study of intelligence, which was the first application of bivariate GCTA, yielded a genetic correlation of 0.62 (0.22) from age 11 to 69.^46^

Thus, the question becomes, why does the heritability of intelligence increase during development despite strong genetic stability from age to age? That is, the same genes largely affect intelligence across the life course and yet genes account for more variance as time goes by. Increasing heritability despite genetic stability implies some contribution from what has been called *genetic amplification.*^57^ This has recently been supported in a meta-analysis of 11500 twin and sibling pairs with longitudinal data on intelligence that found that a genetic amplification model fit the data better than a model in which new genetic influences arise with time.^58^ Genotype-environment correlation seems the most likely explanation in which small genetic differences are magnified as children select, modify and create environments correlated with their genetic propensities. This active model of selected environments—in contrast to the traditional model of imposed environments—offers a general paradigm for thinking about how genotypes become phenotypes.^59^

### Intelligence indexes general genetic effects across diverse cognitive and learning abilities

Another special genetic feature of intelligence is that its differences are caused by genes that affect cognitive abilities as diverse as, for example, spatial ability, vocabulary, processing speed, executive function and memory. Most of the genetic action lies with these general (highly pleiotropic) effects, captured by intelligence, rather than effects specific to each ability, leading to a Generalist Genes Hypothesis.^60^ This is a surprising finding because very different neurocognitive processes appear to be involved in such cognitive abilities.^25^ Although these genetic correlations put intelligence at the pinnacle of the hierarchical model of cognitive abilities mentioned earlier, there is also genetic specificity that builds the genetic architecture for the rest of the hierarchical structure of group factors and specific tests.

In a meta-analysis of 322 studies, the average correlation among individual diverse cognitive tests is about 0.3.^18^ Genetic correlations among cognitive tests are typically greater than 0.6, indicating that the same genes are responsible for the heritabilities of these tests.^60,61^ Genetic correlations estimate the extent to which genetic effects on one trait are correlated with genetic effects on another trait independently of the heritabilities of the two traits. They can be thought about roughly as the probability that genes associated with one trait are also associated with the other trait. Genetic correlations are derived from the genetic analysis of covariance between traits using the same quantitative genetic methods used to analyse variance.^6^

**Figure 2:**
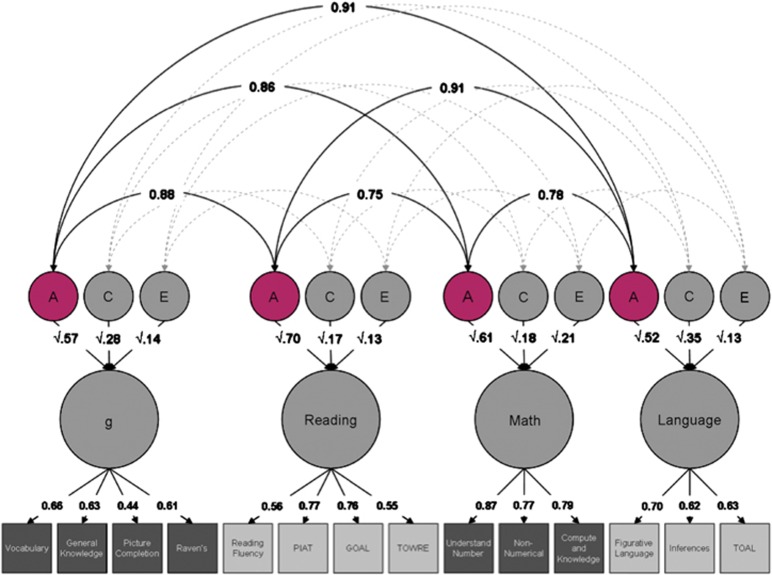
Multivariate (common pathway) genetic analysis in which each latent variable is indexed by three or four tests and the twin method is used to estimate additive genetic (A), shared (common) environmental (C) and nonshared environmental (E) contributions to the variance and covariance among the latent variables. Squares represent measured traits; circles represent latent factors. The lower tier of arrows represents factor loadings; the second tier represents genetic and environmental path coefficients. The curved arrows at the top represent correlations between genetic and environmental latent factors, although only the genetic correlations are shown here. (From Davis *et al.*^62^).

These general genetic effects permeate not only cognitive abilities such as spatial and vocabulary that are used as part of the assessment of intelligence but also extend to education-related learning abilities such as reading and arithmetic. Figure 2 shows the results of a multivariate genetic analysis of 14 tests that comprise four distinct test batteries—intelligence, reading, mathematics and language—for more than 5000 pairs of 12-year-old twins.^62^ The genetic correlations (and 95% confidence intervals) between intelligence and learning abilities are uniformly high: 0.88 (0.84–0.92) with reading, 0.86 (0.81–0.90) with mathematics and 0.91 with language (0.87–0.94). Weighting these genetic correlations by the heritabilities of the latent factors, it can be shown that about two-thirds of the phenotypic correlations between the factors can be explained genetically. One advantage of using such latent factors is that they exclude uncorrelated measurement error. As a result, these genetic correlations are higher than those found when uncorrected composite scores rather than latent factors are analysed: 0.66 (0.05 standard error) for reading, 0.73 (0.03) for mathematics and 0.80 (0.06) for language.^63^

The first attempts to use bivariate GCTA (see Box 1) to verify these twin findings support the hypothesis of general genetic effects on broad cognitive and learning ability-related differences. The GCTA estimates of genetic correlation (and standard error) between intelligence and learning abilities are highly similar to the twin study estimates just mentioned for composite scores uncorrected for error: 0.89 (0.26) for reading, 0.74 (0.15) for mathematics and 0.81 (0.15) for language, estimated from unrelated individuals from the same sample.^63^ Within intelligence, the major group factors of verbal and nonverbal ability yielded a genetic correlation of 1.0 (0.32) in a bivariate GCTA in the same sample.^64^ The high GCTA genetic correlation between verbal and nonverbal based on unrelated individuals supported the twin study estimate of 0.60 (0.09) in the same study.

An important feature of bivariate GCTA is that it yields genetic correlations similar to genetic correlations estimated from the twin method, even though heritabilities are considerably lower for GCTA than for twin estimates. In the study just mentioned, GCTA heritabilities were consistently lower than twin heritabilities: 0.35 vs 0.47 for intelligence, 0.16 vs 0.59 for reading, 0.32 vs 0.48 for mathematics and 0.35 vs 0.41 for language. As noted in Box 1, GCTA heritability estimates are limited to the additive effects tagged by the common single nucleotide polymorphisms (SNPs) used on DNA arrays (i.e., the direct effects of the SNPs on the array and those variants with which they are in linkage disequilibrium); GCTA heritability is lowered by imperfect tagging of causal SNPs. As a result, GCTA heritability estimates are typically about half the heritability estimates from twin studies. This ‘missing GCTA heritability’ is due in part to non-additive effects and the effects of rarer DNA variants. Why then are GCTA estimates of genetic correlation so similar to twin study estimates? The likely reason is that the GCTA estimate of the genetic correlation is derived from the ratio between genetic covariance and the genetic variances of the two traits. Because GCTA’s underestimation of genetic influence applies to genetic covariance as well as to genetic variance, the ratio between genetic covariance and genetic variance cancels out this bias, leaving an unbiased GCTA estimate of genetic correlation.^63^

This finding of strong genome-wide pleiotropy across diverse cognitive and learning abilities, indexed by general intelligence, is a major finding about the origins of individual differences in intelligence. Nonetheless, this finding seems to have had little impact in related fields such as cognitive neuroscience or experimental cognitive psychology. We suggest that part of the reason for this neglect is that these fields generally ignore individual differences.^65,66^ Another reason might be that the evidence for this finding rested largely on the twin design, for which there have always been concerns about some of its assumptions;^6^ we judge that this will change now that GCTA is beginning to confirm the twin results.

This finding of strong genome-wide pleiotropy across diverse cognitive and learning abilities is compatible with multiple neurocognitive models of causal pathways. The modularity model of cognitive neuroscience might suggest that genetic correlations among cognitive abilities are epiphenomenal in the sense that multiple genetically independent brain mechanisms could affect each ability, creating genetic correlations among abilities. However, the genetic principles of pleiotropy (each gene affects many traits) and polygenicity (many genes affect each trait) lead us to predict that generalist genes have their effects further upstream, creating genetic correlations among brain structures and functions, a prediction that supports a network view of brain structure and function.^25,67^

In summary, multivariate genetic research—both from twin studies and GCTA—suggests that most of the genetic action is general across diverse cognitive abilities rather than specific to each ability. Intelligence is a good target for gene-hunting because it indexes these generalist genes.

### Assortative mating is greater for intelligence than for other traits

Although the phenotypic correlation between spouses, assortative mating, might seem an esoteric topic, it has important implications for the genetic architecture of intelligence. Assortative mating is far greater for intelligence than for most other traits. For example, assortative mating is about 0.20 for height^68^ and for weight,^69^ and about 0.10 for personality.^70^ For intelligence, assortative mating is about 0.40.^19,71^ Moreover, verbal intelligence shows greater assortative mating (~0.50) than nonverbal intelligence (~0.30), perhaps because it is easier to gauge someone’s verbal ability such as vocabulary than their nonverbal intelligence such as spatial ability. Assortative mating for intelligence is caused by initial selection of a mate (assortment) rather than by couples becoming more similar to each other after living together (convergence).^72,73^ In part, spouses select each other for intelligence on the basis of education—spouses correlate about 0.60 for years of education^19^—which correlates about 0.45 with intelligence.^50^ Assortative mating may be greater than it is for intelligence for a few other traits such as social attitudes, smoking and drinking, although these traits might be affected by convergence. It should also be noted that not all of the genetic variance for intelligence is additive. For example, dominance, which involves interaction among alleles at a locus, is indicated by research showing inbreeding depression for intelligence.^74^ When assortative mating is taken into account in variance components analysis, some evidence for nonadditive genetic variance emerges.^73,75^

The significance of high assortative mating for intelligence is that assortative mating for polygenic traits increases additive genetic variance. Additive genetic variance refers to the independent effects of alleles or loci that ‘add up’, in contrast to non-additive effects of dominance within a locus, and epistasis across loci in which the effects of alleles or loci interact. Assortative mating of parents increases additive genetic variance in their offspring because offspring receive a random sampling of half of each parent’s genes and resemble their parents to the extent that each allele shared with their parents has an average additive effect. Because offspring inherit only one of each of the parents’ pairs of alleles, offspring differ from their parents for non-additive interactions.

For example, if spouses mated randomly in relation to intelligence, highly intelligent women would be just as likely to mate with men of low as high intelligence. Offspring of the matings of women of high intelligence and men of low intelligence would generally be of average intelligence. However, because there is strong positive assortative mating, children with highly intelligent mothers are also likely to have highly intelligent fathers, and the offspring themselves are likely to be more intelligent than average. The same thing happens for less intelligent parents. In this way, assortative mating increases additive genetic variance in that the offspring differ more from the average than they would if mating were random. The increase in additive genetic variance can be substantial because its effects accumulate generation after generation until an equilibrium is reached. For example, if the heritability of intelligence with random mating were 0.40, the additive genetic variance of intelligence would increase by one-quarter at equilibrium given assortative mating of 0.40, Falconer and MacKay^76^ equation 5, Table 10.6, p. 176.

The extra additive genetic variance for intelligence induced by assortative mating is important for three genetic reasons. First, parents share only additive genetic variance with their offspring, so that genetic predictions from parent to offspring ought to be greater for intelligence when polygenic scores, composite scores based on associations of many loci with intelligence, are available. Second, because GCTA has so far been limited to detecting additive genetic variance, GCTA heritability should be greater for intelligence than for traits that show less assortative mating such as personality. Some evidence supports this prediction in that GCTA heritability estimates for personality appear to be much lower than for intelligence, even taking into account the lower twin-study heritability estimates for personality than for intelligence.^77, 78, 79^ Moreover, GCTA heritability estimates are greater, although not significantly so, for verbal than non-verbal intelligence,^41,80^ which is consistent with the greater assortative mating for verbal than non-verbal intelligence. Third, because both GWA and GCTA are limited to detecting additive genetic variance, the GCTA estimate of substantial additive genetic influence on intelligence makes intelligence a good target for GWA studies.

Two additional points about assortative mating for intelligence warrant mention. First, unlike inbreeding, which reduces heterozygosity across the genome, assortative mating is trait specific—it increases additive genetic variance (changing genotypic frequencies but not allelic frequencies) only for genes associated with the trait for which mates assort and its genetically correlated traits. Second, assortative mating induces a genetic correlation between mates for a particular trait to the extent that the trait is heritable, regardless of whether assortative mating is driven by genetic assortment or by environmental factors such as propinquity. A recent study using genome-wide genotypes showed that spouses are more genetically similar than two individuals chosen at random.^81^ This DNA estimate of genetic similarity between spouses is substantially less than assortative mating for education levels, suggesting that assortative mating may be driven by ‘social sorting processes in the marriage market’.^81^

### Thinking positively: the genetics of high intelligence

Unlike psychiatric and other disorders, intelligence is normally distributed with a positive end of high performance as well as a problematic end of intellectual disability. High intelligence is responsible for exceptional performance in many societally valued outcomes, as documented in long-term longitudinal studies.^82^ Although many other traits, such as those related to athletic performance, are also normally distributed, the importance of high intelligence makes it especially interesting. Genetic exploration of the positive tail of normally distributed traits is important conceptually because it moves away from the notion that we are all the same genetically except for rogue mutations that cause disorders, diseases and disabilities.

Quantitative genetic research on intelligence indicates that the genetic causes of high intelligence are quantitatively, not qualitatively, different from the rest of the distribution. A recent study of 11000 twin pairs found that the top 15% of the intelligence distribution was just as heritable (0.50) as the rest of the distribution (0.55).^83^ Most recently, in a study of 370 000 sibling pairs and 9000 twin pairs in Sweden from 3 million 18-year-old males whose intelligence was assessed as part of compulsory military service, not only was high intelligence (top 4%) just as familial and heritable as the rest of the distribution, a method called DF extremes analysis suggested that the same genetic factors are at work.^84^ DF extremes analysis focuses on the genetic causes of the average difference between an extreme group, however defined, and quantitative trait scores for the population, comparing the differential regression to the population mean for the co-twins of identical and fraternal twin probands.^85^ To the extent that genetics is found to account for this average difference (called ‘group’ heritability), it implies that there is a high genetic correlation between the extreme group and the quantitative trait.^60^ In the Swedish study, DF extremes analysis showed that genetics explained about half of the mean difference between the high-intelligence group and the rest of the distribution, which was similar to the traditional heritability of individual differences and implies strong genetic links between high intelligence and normal variation in intelligence.

It is possible that scores more extreme than the top 4% of the intelligence distribution are aetiologically different from the normal distribution, which has been called the Genetic Discontinuity Hypothesis.^86^ The most persuasive argument for genetic discontinuity for extremely high intelligence was made by David Lykken who noted that a key problem of genius is ‘its mysterious irrepressibility and its ability to arise from the most unpromising of lineages and to flourish even in the meanest of circumstances’ (Lykken^87^ p. 29). Lykken^87, 88^ proposed that genius emerges from unique combinations of genes; he referred to these higher-order nonadditive (epistatic) interactions as *emergenic.* The emergenesis hypothesis does not necessarily predict that different genes affect high intelligence, but it does predict that genetic effects are non-additive for high intelligence. The hallmark of an epistatic trait is one for which identical twins are more than twice as similar as fraternal twins. However, in the two twin studies described above, high intelligence did not show this pattern of twin results and model-fitting analyses found that all genetic influence was additive for high intelligence as well as for the entire distribution of intelligence. Although these results do not support the Discontinuity Hypothesis, the studies were limited to the top 15% and top 4% of the intelligence distribution, which is far short of the extremes of genius, which Galton^89^ benchmarked as the top 0.1%.

The aetiology of high intelligence is also interesting in comparison to intellectual disability. Similar to high intelligence, most intellectual disability is the low end of the normal distribution of intelligence. This has been shown most recently in the Swedish conscript sample mentioned above, with results replicated in a similarly large conscript sample in Israel.^90^ However, extremely severe intellectual disability appears to be aetiologically distinct, as proposed by Lionel Penrose^91^ in 1938 and confirmed in the Swedish and Israeli studies. One critical piece of evidence is that siblings of persons with severe intellectual disability have an average intelligence quotient (IQ) near 100 whereas siblings of persons with mild intellectual disability have an average IQ of about 85, about one standard deviation below the population mean. The absence of genetic links between severe intellectual disability and normal variation in intelligence fits with current molecular genetic research that finds noninherited *de novo* mutations associated with severe intellectual disability.^92^

An hypothesis to integrate these genetic results for the low and high ends of intelligence is this: Normal development of intelligence can be disrupted by any of many mutations including non-inherited *de novo* mutations as well as prenatal and postnatal trauma, but high intelligence requires that everything works right, including most of the positive alleles and few of the negative alleles associated with intelligence. This hypothesis is the rationale for a recent genome-wide case–control association study for cases with extremely high intelligence (IQ>150).^84^ However, one study^93^ has found no association between rare SNPs and intelligence in the normal range of intelligence. In addition, several studies have found no association between copy-number variants, which are typically rare variants, and intelligence in the normal range, although such studies may have been underpowered both in terms of sample and difficulties in assessing copy-number variants.^94^

Although the normal phenotypic distribution of intelligence makes it an obvious target for investigating the high as well as low extremes, the larger significance of positive genetics for psychiatric genetics is that polygenic scores created from GWA studies of psychiatric disorders will be normally distributed, which means that there is a positive end with just as many people as the negative end. This implies that at the level of DNA variation there are no common disorders, only normally distributed quantitative traits.^95^ It also raises the question of who these people are at the positive end of the polygenic distribution of ‘risk’ for psychological and other traits. Are they merely individuals at low risk for problems or do they have special powers? Thinking positively begins by thinking quantitatively—about ‘dimensions’ rather than ‘disorders’ and about genetic ‘variability’ rather than genetic ‘risk’.

### Intelligence brings (some) genetics to ‘social’ epidemiology

It has long been known that intelligence, education and class are correlated. The causes of these associations and their relative contribution to social mobility is much disputed.^96^ Education and social class are also well-established associates of health inequalities, including all-cause mortality.^30^ However, intelligence is a new player in health; its associations with many health and illness outcomes and all-cause and several specific causes of mortality have been discovered in the last decade or so.^97^

We shall explain in this section that, akin to, but broader than cognitive and learning abilities, intelligence shares genetic causes with education and social class, which are touchstone ‘environmental’ variables of diverse social scientists. Major human phenomena studied by these social scientists are social mobility and health inequalities, which are unarguably important. They are studied by sociologists, epidemiologists and economists. Finding out why some people more than others make positive progress in their social position through the life course, and why some people are more prone to illnesses and early death have drafted in the two favourite ‘environmental’ social science variables of education and social class. Education and parental social class are predictors of people’s social position in adulthood.^98,99^ Both, and the person’s own adult social class, are associated with health, illness and mortality: less educated people and those in less professional jobs tend to die earlier.^100, 101, 102, 103^ However, there is a third variable in social mobility research, and a third variable in health inequalities research: intelligence.^104^ Both education and social class are substantially correlated with intelligence.^29,61,105^

Education and social class (which is indexed by occupation, or income, or by the relative deprivation-affluence of where a person lives) are often assumed to be indicators of a person’s environmental influences,^106^ but they are correlated with intelligence, which has a high heritability. Indeed, epidemiologists even use height—shorter stature is associated with earlier mortality—as an indicator of childhood social-environmental influences, though it has high heritability. For example, a recent social epidemiology article described height ‘as a marker of early life insults’.^107^ Here, we emphasise that it is an empirical question rather than something that can be assumed *a priori* as to whether the three key variables in social mobility and health inequalities research—education, social class and intelligence—correlate because of shared genetic and/or environmental causes.

Twin and family studies have shown that educational attainment and social class are somewhat heritable. For example, the pedigree-based estimates of heritability (here as percentages of phenotypic variance explained) in the Generation Scotland family-based study of over 20 000 people were 54% (s.e.=2%) for general intelligence, 41% (2%) for education and 71% (1%) for social deprivation using the Scottish Index of Multiple Deprivation.^108^ The genetic correlation was 0.65 (s.e.=0.02) between intelligence and education, 0.40 (0.02) between intelligence and deprivation and 0.48 (0.02) between education and deprivation. An earlier report on a smaller sample (*N*>6000) of the same study found genetic correlations between intelligence and being physically active outside work (0.25), fruit and vegetable intake (0.23), ever smoking (0.45), smoke exposure (0.53) and income (0.45), with high bivariate heritabilities for all of these.^109^ Another study identified over 2500 pairs of school-age twins from population samples totalling over 300 000 in England and the Netherlands and found moderate to large genetic correlations and bivariate heritability between intelligence and national examination results in language, mathematics and science.^61^ Analyses of older Danish twins found evidence for genetic correlation between cognitive ability and education and health.^110,111^

GCTA studies have recently explored the heritability and genetic correlations of intelligence, education and social class. A combined analysis of Swedish and Australian unrelated subjects (*N*~11 500) used GCTA to provide an estimate of 22% (s.e.=4%) for the heritability of years in education and 25% (8%) for attending college.^16^ In the Twins Early Development Study for 3000 unrelated children, GCTA-based estimates of heritability were 21% (12%) for parental social class and 28% (17%) for children’s IQ at age 7 and 32% (14%) at age 12. The GCTA-estimated genetic correlation between parental social class and IQ was 1.00 (s.e.=0.47) at age 7 and 0.66 (0.31) at age 12.^56^ GCTA-based estimates of heritability on over 6500 unrelated people with genome-wide SNP data in the Generation Scotland study were 29% (5%) for general intelligence, 21% (5%) for education and 18% (5%) for social deprivation.^112^ The genetic correlations were 0.95 (0.13) for intelligence and education, 0.26 (0.16) for intelligence and deprivation, and 0.45 (0.18) for education and deprivation. Therefore, some of the variance in the social scientists’ key environmental variables can be found in DNA variation, some of which is shared with the DNA variation that causes some of people’s differences in intelligence. Another ‘environmental’ social science variable, height, shows a similar set of findings in the Generation Scotland study sample.^108^ The GCTA-estimated heritability of height was 58% (5%), its phenotypic correlation with intelligence was 0.16, the GCTA-based genetic correlation was 0.28 (0.09), and the bivariate heritability was 71%. Bivariate GCTA-derived genetic correlations between intelligence and health variables will require large numbers which are rare, as yet. An analysis of data from the Swedish Twin Registry (*N*=5650 unrelated individuals) found GCTA-derived genetic correlations of 0.13 (s.e.=0.23) and 0.33 (s.e.=0.33) between self-rated health and, respectively, years in education and attending college^16^).

The genetics of intelligence has a special place, therefore, in the heretofore-named ‘social’ epidemiology. Indeed, these new findings from twin/family-based and GCTA-based studies give a corrective to the suggestion that ‘cognitive epidemiology’ be re-named ‘social epidemiology’. Singh-Manoux’s^113^ suggestion was partly made because epidemiologists preferred to use cognitive epidemiology for those studies in which cognition was the outcome, and so there was an objection to Deary and Batty’s (2007)^104^ definition, that is, ‘the use of cognitive ability test scores as risk factors for human health and disease outcomes, including mortality’. Relevant to the genetic associations discussed in this section was Singh-Manoux’s further discussion,

‘Given the association between intelligence and education, extensively discussed by Deary and Johnson,^106^ this definition of cognitive epidemiology puts it squarely in the domain of social epidemiology, a discipline concerned with the social distribution of determinants of health. Location in this broader church, rather than the micro-discipline of cognitive epidemiology, will avoid a narrow focus on intelligence that ignores its associations with markers of social position such as education, income and occupation.’

One might say in reply that this conceptualisation ignores possible genetic contributions to social/cognitive epidemiology. To sum up: there are genetic causes of some of the educational and social class differences in the populations studied, and these overlap with the genetic causes of intelligence differences. Intelligence genetics is special here, because it offers the possibility of finding some of the connections between social and medical outcomes, perhaps *via* genetic contributions to system integrity, allostatic load and the adoption of health-promoting/reducing behaviours.^114^

## Five special findings and polygenic scores

These five special findings about the genetics of intelligence differences have emerged from traditional quantitative genetic research, primarily twin studies, and they are beginning to be replicated using GCTA. However, nothing would advance the field more than moving beyond GCTA to G, C, T, and A—that is, identifying specific DNA variants that contribute to the high heritability of intelligence. As is the case for all complex traits and common disorders in the life sciences, we now know that this will be a difficult task. As discussed earlier, GWA studies have shown that there are no large effect sizes in the population, which implies that the heritability of intelligence is caused by thousands of DNA variants, many of these effects are likely to be infinitesimal or even idiosyncratic. Nonetheless, GCTA has shown that additive effects of common SNPs can theoretically account for at least half of the heritability of intelligence, which means that a brute force approach using ever larger samples will identify some of these genes. In addition, whole-genome sequencing will identify DNA variants of any kind anywhere in the genome, not just common SNPs.

Associations of small effect size between DNA variants and intelligence can be summed across multiple loci to create a polygenic score, which is analogous to aggregating items to create a scale. Polygenic scores can aggregate a few candidate SNPs or thousands of SNPs across the genome, called *genome-wide polygenic scores* (GPS), as described in Box 2.

Anticipating that GPS will be available for research on intelligence, we close by revisiting the five special findings about genetics and intelligence, drawing hypotheses that can tested using a GPS for intelligence, an exercise that we hope will help to make the five special findings more concrete.

### Heritability of intelligence increases dramatically from infancy through adulthood despite genetic stability

GPS hypotheses follow directly from the finding that the heritability of intelligence increases throughout the life course despite strong genetic stability from age to age: Variance explained by a GPS should increase with age, and a GPS discovered at one age, adulthood for example, is expected to predict intelligence at other ages such as childhood.

### Intelligence indexes general genetic effects across diverse cognitive and learning abilities

A GPS hypothesis follows directly from finding strong genome-wide pleiotropy across diverse cognitive abilities: A GPS that is discovered for any cognitive or learning ability should also predict any other ability. Also, a GPS for intelligence should predict better than a GPS for any other trait. It has been suggested that a pleiotropic GPS that explictly targets the substantial covariance among diverse cognitive and learning abilities will be even better than a GPS based on a single composite measure of intelligence.^115^

### Assortative mating is greater for intelligence than for any other trait

GPS support for the previous two hypotheses seems likely because preliminary GCTA results discussed above already provide some support for these hypotheses. In the case of assortative mating, GPS could provide a novel test of the extent to which assortative mating for intelligence is mediated genetically by correlating GPS between spouses. Another question that emerges from previous genetic research is whether GPS assortative mating is greater for verbal than for nonverbal ability.

### Thinking positively: the genetics of high intelligence

Finding that the same genes affect high intelligence to the same extent as the rest of the normal distribution leads to the hypothesis that a GPS for intelligence from unselected samples can also be used to predict high intelligence.

### Intelligence brings (some) genetics to ‘social’ epidemiology

Finding that, in twin and GCTA studies, the same genes influence intelligence and social epidemiologists’ ‘environmental’ variables of education, social class, and height can enlighten research in health and social inequalities. It leads to the hypothesis that GPS scores for intelligence might contribute to health outcomes and mortality, and that these might account for some of the associations between education and class and mortality.

## The first new quantitative genetic method in a century: Genome-wide Complex Trait Analysis (GCTA)

A new method for estimating genetic influence using DNA is a welcome addition to the armamentarium of quantitative genetics. The significance of the method is that it can estimate the net effect of genetic influence using DNA of unrelated individuals rather than relying on familial resemblance in groups of special family members such as monozygotic and dizygotic twins who differ in genetic relatedness. The method is often called GCTA, although its developers refer to it as Genomic-Relatedness-Matrix Restricted Maximum Likelihood.^32, 33, 34, 35^ Other methods^36^ and modifications^37,38^ are also emerging.^39,40^

Like other quantitative genetic designs such as the twin design, GCTA uses genetic similarity to predict phenotypic similarity. However, instead of using genetic similarity from groups differing markedly in genetic similarity such as monozygotic and dizygotic twins, GCTA uses genetic similarity for each pair of unrelated individuals based on that pair’s overall similarity across hundreds of thousands of single nucleotide polymorphisms (SNPs) for thousands of individuals; each pair’s genetic similarity is then used to predict their phenotypic similarity. Even remotely related pairs of individuals (genetic similarity greater than 0.025, which represents fifth-degree relatives) are excluded so that chance genetic similarity is used as a random effect in a linear mixed model. The power of the method comes from comparing not just two groups like monozygotic and dizygotic twins, but from the millions of pair-by-pair comparisons in samples of thousands of individuals. In contrast to the twin design, which only requires a few hundred pairs of twins to estimate moderate heritability, GCTA requires samples of thousands of individuals because the method attempts to extract a small signal of genetic similarity from the noise of hundreds of thousands of SNPs. A handy power calculator is available, which underlines the large samples needed for GCTA (http://spark.rstudio.com/ctgg/gctaPower/).

GCTA detects only those genetic effects tagged by the common SNPs (allele frequencies typically much greater than 1%) that have until recently been incorporated in commercially available DNA arrays used in GWA studies. This limitation is changing as exome arrays became available in 2013 that included rare SNPs in or near exomes (http://res.illumina.com/documents/products/datasheets/datasheet_human_core_exome_beadchip.pdf); the limitation will be lifted as whole-genome sequencing is more widely used. In addition, GCTA is limited to detecting the additive effects of SNPs; it cannot detect gene–gene or gene–environment interaction. Thus, GCTA heritability represents the upper limit for detection of SNP associations in GWA studies, which, like GCTA, are limited to detecting additive effects of common SNPs. Conversely, GCTA heritability represents the lower limit for heritability estimated in twin studies because twin studies can detect genetic influence due to DNA variants of any kind. In this way, the comparison between GCTA and twin study estimates of heritability reveals fundamental information about the genetic architecture of complex traits, including intelligence.

Similar to other complex traits, GCTA heritability estimates for intelligence are about half the heritability estimates from twin studies.^6,41^ This finding suggests that despite the modest yield so far from GWA studies of intelligence,^14^ with sufficiently large samples, it should in theory be possible to detect as much as half the heritability with the additive effects tagged by the common SNPs on currently available DNA arrays. The missing heritability gap between GCTA and twin studies is likely to be filled in part by less common DNA variants which will be detected as whole-genome sequencing comes on line.^42^

The value of GCTA has been greatly increased by extending it beyond the univariate analysis of the variance of a single trait to the bivariate analysis of the covariance between two traits or the ‘same’ trait at two ages;^43,44^ a recent approach is multivariate rather than just bivariate.^45^ Bivariate GCTA was first applied to intelligence, yielding a high genetic correlation between intelligence scores in childhood and old age,^46^ as described in the text along with other examples of bivariate GCTA.

## Polygenic Scores

There are at least a dozen labels to denote such polygenic scores, most of which involve the word ‘risk’, such as *polygenic risk scores,* but we prefer the term *polygenic score* because it makes more sense for quantitative traits like intelligence with positive as well as negative poles.^115^ A polygenic score is created by adding genotypic values across loci. For example, for one locus (A) with two alleles (A1 and A2), with the A2 allele associated with higher intelligence scores, additive genotypic values can be assigned for locus A so that A1A1 individuals=0, A1A2 individuals=1 and A2A2 individuals=2. For each individual, these 0, 1 and 2 additive genotypic values for locus A can be added to those for locus B to create a polygenic score that varies from 0 to 4, and so on for dozens, hundreds or thousands of loci. A refinement is to weight each loci by the strength of its association with intelligence. For example, 100 associations that each account for 0.1% of the variance of intelligence on average could together account for 10% of the variance. Their effects should add up because DNA variants are uncorrelated unless they are very close together on a chromosome. Any loci could be aggregated to create a polygenic score, such as candidate genes thought to be associated with a trait. However, the most productive use of polygenic scores is to aggregate genotypic scores for DNA variants (usually SNPs) known to be associated with a trait. For example, much research on body weight has used a polygenic score based on 32 SNPs that have shown replicated associations with body mass index, even though this polygenic score only accounts for 2% of the heritability of body mass index.^116^

A more recent variant of polygenic scores goes beyond aggregating a few dozen individual SNPs associated with a trait to include thousands of SNPs from GWA studies in a *genome-wide polygenic score* (GPS) that includes thousands of SNPs or even all SNPs on a DNA array weighted by the strength of their association.^115,117^ The idea is that a GPS will be enriched for positive associations even though the GPS will certainly include false-positive associations. Although GPS can theoretically account for all the heritability shown in GCTA,^118^ GWA studies of intelligence and other traits have resulted in GPS that fall far short of GCTA estimates of heritability. For intelligence in childhood, a GPS derived from a discovery sample of more than 12 000 children accounted for about 2% of the variance in independent samples of more than 5000.^14^ For intelligence in adulthood, about 1% of the variance was explained by a GPS derived from an adult sample of 3200 individuals and tested in an independent sample of 670 individuals,^80^ even though GCTA estimates of heritability are about 30%. Using years of education rather than intelligence *per se*, the meta-analysis mentioned earlier with 125 000 individuals in a discovery sample yielded a GPS that accounted for 2 and 3% in two independent samples.^16^ More variance in intelligence is likely to be explained with GPS derived from larger samples, whole-genome sequencing and more novel strategies such as using networks of functionally linked genes.^119^

Having a GPS for intelligence that reliably accounts for even as little as a few percent of the variance will enhance research on intelligence. It will enable DNA analyses at the level of individuals rather than families which can address the major questions of quantitative genetic research such as developmental, multivariate and GE interplay issues mentioned earlier. Polygenic scores can be used in the same way that candidate genes have been used. A neuroscientist might not find a polygenic score useful for investigating molecular pathways between genes and behaviour through the brain, except perhaps to emphasise the need for a network approach governed by pleiotropy (each gene affects many traits) and polygenicity (each trait is affected by many genes). A GPS for intelligence would be like the other GPS (global positioning system) making it possible to triangulate on the genetics of intelligence from all domains of the life sciences, for example, integrating research on the genetics of intelligence from the genome, epigenome, transcriptome, proteome and metabalome to the brain and behaviour without the need to assess intelligence.^115^
